# Factors responsible for dropout in the Ponseti method of clubfoot treatment: A cross-sectional study

**DOI:** 10.5339/qmj.2024.63

**Published:** 2024-11-11

**Authors:** Manzoor, Alam Zeb, Ubaid Ullah, Arif Shah

**Affiliations:** 1 Physical Therapist at Islamabad Club, Islamabad, Pakistan *Email: manzoorayaz68@gmail.com; 2 Department of Physiotherapy & Rehabilitation, Hasselt University, Hasselt, Belgium; 3 Clubfoot Department, Lady Reading Hospital, Peshawar, Pakistan; 4 Physical Therapist, Paraplegic Center Peshawar, Pakistan

**Keywords:** Dropout, Ponseti method, barriers, clubfoot

## Abstract

**Background:**

The Ponseti method for clubfoot treatment is a non-surgical treatment with a successful outcome. However, many children are not able to complete Ponseti treatment due to various barriers and are termed dropout children. This study aimed to find out the factors responsible for dropout from clubfoot treatment in Peshawar, Pakistan.

**Methods:**

The study was conducted in the Clubfoot Department of a tertiary care hospital. Clinical and demographic information like age, casting phase, bracing phase, and clubfoot types, i.e., idiopathic, syndromic, or neurogenic, were extracted from the hospital database, while for dropout factors, a semi-structured questionnaire was used. Descriptive statistics were applied to demographic data, assessments, and semi-structured questionnaires for parents. The association of dropout rates with age, gender, and unilaterally/bilaterally clubfoot was analyzed through chi-square tests.

**Results:**

Between December 2017 and December 2022, a total of 1,150 babies were treated with the Ponseti method in the Clubfoot Department. Of 1,150 patients, 197 (17.1%) patients dropped out of the treatment. Twenty-four (12.1%) patients of this dropout were from the casting phase, and 173 (87.9%) patients were from the bracing phase. Age was found to be a significant factor affecting dropout from the Ponseti method. No significant association was found between the patient’s dropout and gender or between dropout and unilaterally/bilaterally clubfoot.

**Conclusion:**

The Ponseti method improves clubfoot treatment but faces high dropout rates due to barriers like lack of family support and transport issues. Clinics addressed this by offering family support, transportation aid, telemedicine, community outreach, financial aid, peer support, and extended hours, reducing dropout rates and enhancing outcomes.

## 1. Introduction

Congenital Talipes Equino-Varus (CTEV), commonly known as clubfoot, is a prevalent orthopedic abnormality seen in pediatric patients.^1^ Clubfoot affects approximately 1.24 per 1,000 births and demonstrates prevalence across all six continents. It is observed that males are affected more frequently than females, with a ratio of 2:1.^2^ Eighty percent (90%) of these babies are born in Low and Middle-Income Countries (LIMCs) in which less than 15% receive proper treatment.^2^ The causes of clubfoot can be categorized into idiopathic, syndromic, or neurogenic. Idiopathic Clubfoot typically lacks a clear underlying cause and is considered to be of unknown origin. Syndromic Clubfoot occurs as part of a larger syndrome or genetic condition. Neurogenic Clubfoot is associated with neurological disorders or abnormalities affecting the muscles and nerves of the lower leg and foot.^3^ Without appropriate management, babies with clubfoot may face life-long deformity, disability, and social stigma, which may hamper their access to proper education and employment.^1^ Due to its effectiveness, the Ponseti method is considered the most broadly accepted treatment option among the non-surgical treatments for clubfoot, with 68–95% success ratio.^4–8^ Dr. Ignacio Ponseti revolutionized clubfoot treatment with his non-surgical method, offering effective care to children worldwide. The Ponseti method is composed of two steps: casting, including tenotomy, and bracing phase. In the casting phase, the foot is gradually adjusted to the correct position, while the bracing phase ensures it remains in place. This process typically begins when a baby is around one to two weeks old, during which they undergo a series of 5–7 casts over several weeks or months. Once the foot reaches its final, proper alignment, the baby is then provided with a brace for ongoing support.^8^ However, this method faces a lot of challenges, especially in those countries with limited resources, such as delayed presentation, limited access to healthcare services, loss to follow-up, non-compliance, and high relapse rates.^1,2,4,5^ The success of the Ponseti method is dependent upon proper treatment and regular follow-ups to prevent relapse. The Ponseti method requires the clubfoot patients to follow the treatment protocols and visit the treatment center for a prolonged period (4–5 years) if the risk of relapse is very high.^2,9^

A lot of literature is available on the efficacy of the Ponseti method and factors connected with relapse clubfoot after the Ponseti method.^10–12^ However, a few studies have focused on the dropout rates during and after the Ponseti method.^3,13^ The reasons behind this dropout in LMICs are poverty, lack of access to health care, lack of information, and social and cultural factors.^14–17^

Dropout during the casting and tenotomy phases of the Ponseti method will lead to insufficient correction of the foot and residual abnormality, while dropout during the Foot Abduction Brace (FAbB) of the Ponseti method may be connected with a high risk of relapse.^2,9^ If the factors responsible for dropout can be stopped, more babies will complete the Ponseti method of treatment, which is both economical and highly effective.^18^ Therefore, it is important to understand the reasons for patients’ dropout from the Ponseti method because these dropout patients will gradually move towards disabilities and will suffer socially and economically in the future. This study aimed to find out the factors responsible for the dropout of the Ponseti method in Peshawar, Pakistan.

## 2. Methods

The study was conducted in the Clubfoot Department of a tertiary care hospital in Peshawar. The study was ethically approved by Khyber Medical University (DIR/KMU-ASRB?PR/001967) and Lady Reading Hospital Physiotherapy Department, Peshawar. The Ponseti method is considered as a standard care of treatment in this department. The study design was cross-sectional. It was conducted in Clubfoot Department from December 2017 to December 2022. During this period, the Ponseti method was used to treat clubfoot cases. Clubfoot was diagnosed through a comprehensive process involving a thorough physical examination and X-rays. The severity of the deformity was categorized using the Pirani score.^19^ This approach ensured accurate diagnosis and informed appropriate treatment strategies for patients with clubfoot. According to the International Clubfoot Registry (ICR), a dropout child was a child that had not visited within three weeks of casting (Casting Dropout), within four weeks of tenotomy (Tenotomy Dropout), or within six weeks of the last visit for those whose on bracing phase (Bracing Dropout).^20^ Babies that fit into one of the above definitions were recognized as a dropout. The inclusion criteria were based on the definition of dropout. Clinical and demographic information like age, casting phase, bracing phase, and clubfoot types, i.e., idiopathic, syndromic, or neurogenic, were extracted from the hospital database, while for dropout factors a semi-structured questionnaire was used based on the Social Ecological Model prepared and used by Pinto et al.^13^ Parents or guardians of eligible patients were contacted and provided with comprehensive information about the study. Those who agreed to participate signed an informed consent form prior to enrollment. The questionnaire was filled out by an independent interviewer from the patient’s caregivers/parents through mobile phones and physically by invitation to the Clubfoot Department.

After collection, the data were entered into SPSS version 22 for further analysis. Descriptive statistics were applied to the demographic data, assessments, and semi-structured questionnaires for parents. The association of dropout rates with age, gender, and unilaterally/bilaterally clubfoot was evaluated through chi-square tests.

## 3. Results

**Figure 1.**
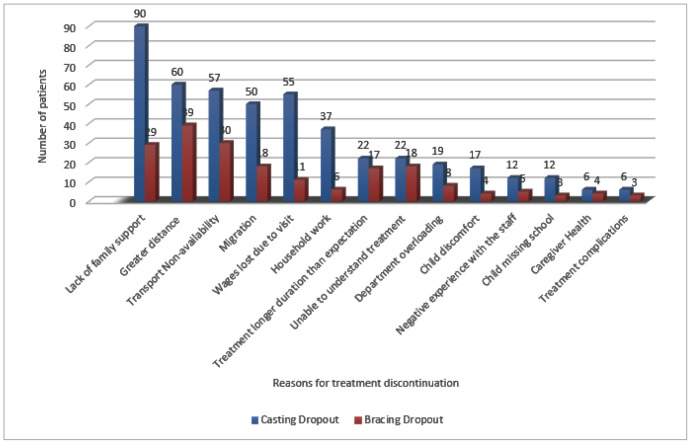
Reasons cited by parents for discontinuation of clubfoot treatment at the Lady Reading Hospital, Peshawar, from December 2017 to December 2022.

Between December 2017 and December 2022, a total of 1,150 babies were treated with the Ponseti method in the Clubfoot Department. Of 1,150 patients, 197 (17.1%) patients dropped out of the treatment. Twenty-four (12.1%) patients of this dropout were from the casting phase, and 173 (87.9%) patients were from the bracing phase. For demographic characteristics of 197 dropout patients and 953 regular patients, see Table 1. Age was found to be a significant factor affecting dropout from the Ponseti method. Statistical significance was present in the age of dropout patients as compared to regular follow-up (chi-square value is 16.9, p-value is 0.002). No significant association was found between patient dropout and gender, nor between dropout and unilaterally/bilaterally clubfoot (Table 1). Of 197 dropouts, 156 parents were contacted through mobile numbers while the remaining 41 were unable to contact due to non-availability/changing of contact number. Of these 156, 135 (87.7%) dropped out during the bracing phase, and 21 (13.3%) dropped out during the casting phase. The reasons cited by the parents are listed in Figure 1.

## 4. Discussion

The Ponseti is considered a gold standard treatment for clubfoot. It is a minimally invasive, cost-effective method that is linked with the best clinical outcomes as compared to the surgical method.^5,21,22^ However, the method faces growing concerns about patients’ loss of ability to follow up on the treatment. This could affect the treatment outcomes, more importantly in the lower socioeconomic countries. Numerous studies have observed these barriers that are faced by the service providers and caregivers in the delivery of the Ponseti method in LMICs in Asia, Africa, and Latin America.^3,6,16,17,23,24^ The dropout rate from the Ponseti treatment ranges from 14 to 41%.^3,13,25–27^ The dropout rate in our study was 197 (17.1%) out of 1,150 patients, which is similar to the above-mentioned studies.

Various barriers to the Ponseti method and dropout clubfoot, identified by different studies in LMICs, are poverty, lack of family support, domestic issues, lack of transport, clinic being overcrowded, no proper training of the physical therapist, unable to understand clinician advice, caregivers other responsibilities, travel expenses, etc.^1,3,13,28^ Our study showed that various factors responsible for the dropout were similar to the above-mentioned literature, such as lack of family support (76.2%), distance to hospital (63.4%), non-availability of transport (55.7%), etc.

Age is considered an important factor for Ponseti method successful outcomes and dropout rates.^1^ The children who are presented at an older age need a greater number of cast changes, thus increasing the cost of the treatment. Similarly, older children are more active as compared to infants and may not be able to tolerate casts and braces. This may increase the chances of dropout from the treatment.^9,13,29^ Dropouts consistently occurred during the bracing phase, typically when children were over two years old and already walking. Parents faced numerous competing demands, compounded by a lack of awareness regarding the importance of the nighttime brace, a commonly expressed regret in hindsight. Challenges such as travel distances, financial constraints, time constraints due to work and familial responsibilities, emotional distress, and social stigma surrounding their child’s clubfoot deformity contributed to treatment discontinuation for some parents.^3^ This statement is supported by our study. This study showed that the rate of dropout was greater in older children as compared to infants. The dropout rate was greater during the bracing phase (87.9%) as compared to the casting phase (12.15%), because older children were not able to tolerate the brace for a long time. Factors contributing to this discrepancy included the duration of treatment, discomfort issues associated with wearing the brace, adherence to the treatment plan, and ongoing support and education provided to families during the bracing phase.

Surprisingly, female gender is not found to be a significant factor in the dropout rate. Traditionally, the female gender is always discriminated against male in LIMCs. Female babies are more likely to start treatment at a later stage as compared to male babies.^1,3,30^ Behera et al. found that more boys dropped out during the casting phase of the treatment compared to girls. Similar results were found by Pinto et al., but they also found more boys dropping out than girls over the entire course of the treatment. However, it’s important to note that neither the results of Behera et al.^31^ nor the results of Pinto et al.^13^ were found to be significant. Our study showed a high rate of dropout among boys during the casting phase and throughout the entire course of the treatment. However, our study showed no statistically significant association between gender and dropout rates.

There is no clear definition of dropout in the existing literature. According to the clubfoot training resource, “Dropout depends upon the phase of the treatment.” A child in the casting phase misses more than two weeks could be considered a dropout, or a child in the bracing phase for six months who does not attend for nine months could be considered a dropout.^32^ The ICR has defined three types of dropouts as follows: Casting dropouts have not had a visit within three weeks of the given date; Tenotomy dropouts have not come for a visit within four weeks of the end date, and Bracing dropout has not a visit within six months of the end date (end date means the last schedule appointment).^20^ Both definitions are time-bound and cannot be applied globally. Similarly, we defined dropout as those patients who had missed two or more frequent follow-up appointments either in the casting phase or the bracing phase.

The study had a few limitations. One of them was a small sample size and single-center study. The other one was the 41 patient’s parents, who were not interviewed despite several attempts to contact them.

Some of the recommendations from the dropout parents were financial support for travel, appointment reminders, improved clinicians’ attitudes and manners, providing treatment at district-level hospitals, etc. Three obstacles have been identified, namely departmental overload, negative experiences with staff, and difficulty understanding the treatment plan. These challenges are directly related to the practice. To address them, plans for change may include implementing measures to streamline departmental workflows, improving staff training and communication skills to foster positive interactions, and enhancing patient education efforts to ensure clarity in treatment plans. By addressing these obstacles, the practice aims to enhance the overall patient experience and improve treatment outcomes.

## 5. Conclusion

The Ponseti method is a non-invasive procedure with better outcomes than surgical treatment. However, the dropout rate from the treatment is the main hurdle to the Ponseti method. This dropout is due to various barriers like lack of family support, distance and access to the treatment facility, etc. To address the hurdles contributing to dropout rates, the clinic could establish family support programs to educate families on treatment benefits, provide transportation assistance, introduce telemedicine services, conduct community outreach programs, offer financial support, facilitate peer support groups, and extend clinic hours.

## Acknowledgment

I would like to express my deepest gratitude to the Physiotherapy Department of Lady Reading Hospital, Peshawar, for their support throughout the project.

## Figures and Tables

**Table 1.** Demographic and clinical characteristics of clubfoot patients at the Lady Reading Hospital Clubfoot Department, Peshawar, from December 2017 to December 2022.

**Variables**	**Regular follow-up**	**Casting dropout**	**Bracing dropout**
Age at first casting (In Months)	2.17 ± 1.60	3.21 ± 3.10	3.90 ± 3.30
Types of clubfoot			
Idiopathic	792 (83.1%)	17 (70.8%)	148 (85.5%)
Syndromic	110 (11.6%)	4 (16.6%)	19 (10.9%)
Neurogenic	53 (5.3%)	3 (12.6%)	6 (3.5%)
Foot/feet affected			
Bilateral	333 (34.9%)	17 (70.8%)	129 (74.5%)
Unilateral	620 (65.1%)	6 (30.2%)	44 (25.5%)
